# Primaquine Clears Submicroscopic *Plasmodium falciparum* Gametocytes that Persist after Treatment with Sulphadoxine-Pyrimethamine and Artesunate

**DOI:** 10.1371/journal.pone.0001023

**Published:** 2007-10-10

**Authors:** Seif Shekalaghe, Chris Drakeley, Roly Gosling, Arnold Ndaro, Monique van Meegeren, Anders Enevold, Michael Alifrangis, Frank Mosha, Robert Sauerwein, Teun Bousema

**Affiliations:** 1 Department of Medical Microbiology, Radboud University Nijmegen Medical Centre, Nijmegen, The Netherlands; 2 Kilimanjaro Christian Medical Centre, Moshi, Tanzania; 3 Department of Infectious and Tropical Disease, London School of Hygiene and Tropical Medicine, London, United Kingdom; 4 Joint Malaria Programme, Kilimanjaro Christian Medical Centre (KCMC), Moshi, Tanzania; 5 Institute for International Health, Immunology and Microbiology, Center for Medical Parasitology (CMP), University of Copenhagen, Copenhagen, Denmark; World Health Organization, Switzerland

## Abstract

**Background:**

*P. falciparum* gametocytes may persist after treatment with sulphadoxine-pyrimethamine (SP) plus artesunate (AS) and contribute considerably to malaria transmission. We determined the efficacy of SP+AS plus a single dose of primaquine (PQ, 0.75 mg/kg) on clearing gametocytaemia measured by molecular methods.

**Methodology:**

The study was conducted in Mnyuzi, an area of hyperendemic malaria in north-eastern Tanzania. Children aged 3–15 years with uncomplicated *P. falciparum* malaria with an asexual parasite density between 500–100,000 parasites/µL were randomized to receive treatment with either SP+AS or SP+AS+PQ. *P. falciparum* gametocyte prevalence and density during the 42-day follow-up period were determined by real-time nucleic acid sequence-based amplification (QT-NASBA). Haemoglobin levels (Hb) were determined to address concerns about haemolysis in G6PD-deficient individuals.

**Results:**

108 individuals were randomized. *Pfs25* QT-NASBA gametocyte prevalence was 88–91% at enrolment and decreased afterwards for both treatment arms. Gametocyte prevalence and density were significantly lower in children treated with SP+AS+PQ. On day 14 after treatment 3.9% (2/51) of the SP+AS+PQ treated children harboured gametocytes compared to 62.7% (32/51) of those treated with SP+AS (p<0.001). Hb levels were reduced in the week following treatment with SP+AS+PQ and this reduction was related to G6PD deficiency. The Hb levels of all patients recovered to pre-treatment levels or greater within one month after treatment.

**Conclusions:**

PQ clears submicroscopic gametocytes after treatment with SP+AS and the persisting gametocytes circulated at densities that are unlikely to contribute to malaria transmission. For individuals without severe anaemia, addition of a single dose of PQ to an efficacious antimalarial drug combination is a safe approach to reduce malaria transmission following treatment.

**Trial Registration:**

Controlled-Trials.com ISRCTN61534963

## Introduction

The majority of anti-malarial drug treatments target the asexual blood stages of *Plasmodium falciparum* that are responsible for clinical disease and death. Sexual stage parasites, gametocytes, can also be present in infected individuals and are responsible for the transmission of the parasite to mosquitoes. Drugs specifically targeting these sexual stage parasites may affect the spread of malaria in the human population. Anti-gametocyticidal drugs are used by several countries to prevent onward transmission from clinical malaria cases [1] and have also been evaluated by mass drug administration to reduce malaria transmission in communities [2].

Artemisinin-based combination therapies (ACT) are advocated as first-line antimalarial treatment because of their high treatment efficacy [3]–[7] and beneficial effects on malaria transmission [4]–[7]. Although ACT efficiently reduces microscopic levels of gametocytes [4]–[7], submicroscopic gametocytes (detected by molecular analysis) may persist after treatment and allow post-treatment malaria transmission [4]. The implementation of ACT may have a beneficial influence on malaria transmission in the general population [8] but ACT may not be sufficient to completely prevent post treatment malaria transmission. Primaquine (PQ) may be of added value in attempts to block malaria transmission as part of a mass drug administration. PQ is an 8-aminoquinolone that is widely used for the treatment of *P. vivax* malaria and actively clears mature *P. falciparum* gametocytes [9]–[11]. Although there is no consensus about which drug is the most potent gametocytocidal drug [10], [11], artesunate (AS) may predominantly inhibit gametocyte development while PQ may accelerate gametocyte clearance [11]. In combination with sulphadoxine-pyrimethamine (SP) and AS, PQ was found to be safe and highly efficacious in clearing asexual parasites and *P. falciparum* gametocytes detected by microscopy [12]. The efficacy of this combination on submicroscopic gametocytaemia is unknown and, in general, information on PQ use in Africa is scarce. Prior to the wide-scale introduction of ACT, a single dose of PQ following first line antimalarial treatment was recommended by the World Health Organisation to reduce malaria transmission in low endemic areas [1]. Although several countries adopted this recommendation, there is concern for negative haemolytic side effects in individuals who are glucose-6-phosphate-dehydrogenase (G6PD) deficient [1], [10].

Here, we determine the safety and efficacy of SP+AS plus a single dose of PQ on clearing submicroscopic levels of *P. falciparum* gametocytaemia in an area of hyperendemic malaria in north eastern Tanzania. Possible haemolytic effects of PQ were determined in relation to G6PD status.

## Methods

The protocol for this trial and supporting CONSORT checklist are available as supporting information; see Checklist S1 and Protocol S1. The trial was registered at Current Controlled Trials; ISRCTN61534963; http://www.controlled-trials.com/ISRCTN61534963/. Registration was done after patient recruitment started due to communication problems.

### Participants

This study was conducted in the period July through September 2006 in Mnyuzi, a rural village in the Tanga Region, north eastern Tanzania. Malaria transmission intensity is high with an estimated entomological inoculation rate (EIR) of 91 infectious bites per person per year [13]. The rainfall pattern is bimodal, with a long rainy season between March and June, and a short rainy season between October and December. The study protocol was approved by the ethics committees of Kilimanjaro Christian Medical Centre, the Tanzanian National Institute for Medical Research (NIMR/HQ/R.8a Vol. XIII/446) and the London School of Hygiene and Tropical Medicine (#4097). Participants were recruited from among children consulting the Mnyuzi health centre and who were resident within a 10 kilometres radius. Informed consent was obtained form the child's parents or guardians prior to inclusion. Children aged 3–15 years with a temperature >37.5C° or a history of fever within the last 48 hours and with *P. falciparum* mono-infection at a density between 500–100,000 parasites/µL were eligible for recruitment. Exclusion criteria were: a haemoglobin (Hb) concentration measured by Hemocue® below 8g/dL, inability to take drugs orally, known hypersensitivity to any of the drugs given, reported treatment with antimalarial chemotherapy in the past 2 weeks, evidence of chronic disease or acute infection other than malaria, domicile outside the study area, signs of severe malaria and eligibility for other malaria studies conducted in the region.

### Interventions

Participants enrolled were randomized to one of the two treatment regimes:

- Sulphadoxine (25 mg/kg) and pyrimethamine (1.25 mg/kg) as a single dose (SP; Fansidar®, Roche, Switzerland) *plus* artesunate (AS), 4 mg/kg once daily for three days (Arsumax®, Sanofi-aventis, France) *plus* placebo once on the third day (Organon, The Netherlands);
- SP *plus* AS *plus* primaquine (base; department of clinical pharmacology, Radboud University Nijmegen Medical Centre, the Netherlands) as a single dose on the third day (0.75 mg/kg). Primaquine capsules were produced following regulations of the European Pharmacopeia.

Treatment was administered by staff at the recruitment clinic. Each child was observed for 30 minutes after treatment, a replacement dose was given in case of vomiting. None of the children had repeated vomiting. Paracetamol (10 mg/kg) was given until symptoms had subsided. In case of parasitological treatment failure, rescue treatment with mefloquine was administered (Lariam®, Roche, Switzerland; 15 mg/kg on first day and 10 mg/kg on second day). All staff engaged in the trial were blinded as to the treatment group of each child, apart from the study physician who administered medication.

### Objectives

Our primary objectives were to determine the effect of SP+AS and SP+AS+PQ on submicroscopic *P. falciparum* gametocyte prevalence and density and to determine the safety of a single dose of PQ in glucose-6-phosphate-dehydrogenase (G6PD) deficient children.

### Outcomes

The primary outcomes were gametocyte prevalence and density by real-time nucleic acid sequence-based amplification (QT-NASBA). The secondary outcome was haemoglobin concentration following treatment. Other outcomes that we evaluated were microscopic gametocyte prevalence, treatment efficacy and the occurrence of side effects.

Participants were encouraged to attend the recruiting clinic at day 1, 2, 3, 7, 14, 28 and 42 after enrolment and at any time the child became unwell. On each day of follow-up, tympanic temperature was measured by electric thermometer and a finger prick blood sample was used for haemoglobin (Hb) measurement using a Hemocue photometer (Angelholm, Sweden), a microscopic slide, a 50 µL-blood sample for real-time nucleic acid sequence-based amplification (QT-NASBA) and a filter paper sample. The presence of symptoms suggestive of anaemia (fatigue, weakness, dizziness, headache, heart palpitations) or allergic drug reactions (rash) was assessed verbally during every follow-up visit. Field assistants visited the homes of children who failed to show up to collect additional samples.

Blood smears were stained for 10 minutes with 10% Giemsa and screened for asexual parasites and gametocytes at enrolment and on day 3, 7, 14, 28 and 42 after treatment. All slides were double-read by experienced microscopists and were declared negative if no parasites were observed in 100 microscopic fields. Readings were compared for validation and slides giving discordant results were read by a third reader. The majority result was taken as final in the case of positive versus negative results and geometric mean of the two closest values for density discordants . Asexual parasites and gametocytes were counted against 200 and 500 white blood cells, respectively and converted to parasites/µL by assuming a density of 8000 white blood cells/µL blood.

*P. falciparum* parasite detection by QT-NASBA was performed as described previously [14], [15]. Briefly, nucleic acids were extracted from 50 µL-blood samples with initial RNA extraction carried out in the field following the original Guanidine isothiocyanate (GuSCN) RNA extraction method [16] until the nucleic acids were bound to silica dioxide particles. At this point, samples were stored at −20°C prior to completion of the extraction and QT-NASBA analysis. QT-NASBA was performed on a NucliSens EasyQ analyser (bioMérieux, Boxtel, the Netherlands) for Pfs25 mRNA. The Pfs25 QT-NASBA is gametocyte-specific and has a detection limit of 10–100 gametocytes/mL. Nuclisens Basic kits (bioMérieux, Boxtel, the Netherlands) were used for amplification according to the manufacturers instructions. A standard dilution series of *in vitro* cultured mature NF54 gametocytes was included in each run to ascertain gametocyte density [17].

### Molecular Genotyping

#### DNA extraction and PCR genotype analysis

DNA extraction from bloodspots on filter paper was carried out by the chelex-100 method as described by Wooden *et al*. [18] with some modifications described in Pearce *et al.* [19]. In brief, all samples were extracted in a 96-well plate format. The bloodspot was first soaked in phosphate-buffered saline (PBS) with 0.5% saponin overnight and was then washed twice in 1 ml of PBS. The samples were then boiled for 8 min in 100 µL of H_2_O and 50 µL of 20% chelex suspension in distilled water (pH 9.5) and centrifugated at 5000 rpm for 10 minutes. 1 µL of supernatant was used in the PCR reactions.

#### Genotyping for MSP-1, MSP-2 and glucose-6-phosphate-dehydrogenase (G6PD) deficiency

To differentiate between recrudescent parasites i.e. those persisting from the initial infection and parasites from a new infection, a nested PCR amplification of the polymorphic regions of *P. falciparum* genes msp1 (K1, MAD20 and RO33) and msp2 (IC1 and FC27) was performed as described by Snounou *et al* [20]. This PCR was performed for follow-up samples with microscopically confirmed parasitaemia. The PCR products (10 µL) were run in electrophoresis on 2–2.5% metaphor agarose gels in 1xTBE buffer, stained with ethidium bromide and then visualized in UV trans-illumination. The procedure of Cattamanchi *et al*. was followed in that indeterminate samples for which a majority of novel bands appeared for the post-treatment infection were scored as new infections [21].

G6PD deficiency was determined by screening human DNA for single nucleotide polymorphisms in the G6PD gene (G202A, A376G) by a simple high throughput method using PCR, sequence specific oligonucleotide probes (SSOPs) and ELISA-based technology [22]. Individuals with no G202A mutation were classified G6PD B, heterozygotes for the G202A mutation were classified G6PD A and homozygote or hemizygote (males) for the G202A mutation were classified G6PD A-.

### Sample size

The primary endpoint used for sample size calculation was *Pfs25* QT-NASBA gametocyte prevalence after treatment. Assuming a gametocyte prevalence in the SP+AS group of 50% on day 14 after treatment [4], a sample size of 50 individuals per group would allow over a power of 80% to detect a reduction in gametocyte prevalence to 20% in the SP+AS+PQ group, allowing for 5% drop-out and using a significance level of 0.05. This sample size also allowed us to detect a two-fold reduction in gametocyte prevalence during the entire period of follow-up in longitudinal data analyses, assuming an average *Pfs25* QT-NASBA gametocyte prevalence of 58% in SP+AS treated children [4], and a maximum correlation between observations of the same individual of 0.30.

### Randomization

The randomization sequence was generated in Stata 8.0 (Stata Corporation, Texas, USA) using restricted randomization with a block size of 20. Treatment allocation was determined by opening pre-prepared randomization envelopes in sequence by the study physician. The same physician was involved in participant selection and clinical evaluation. Parasite carriage by microscopy and *Pfs25* QT-NASBA and haemoglobin concentrations were determined by technicians who were unaware of the treatment allocated to study participants.

### Statistical methods

Therapeutic outcome was classified as early parasitological treatment failure (ETF), late treatment failure (LTF), re-infection or adequate clinical and parasitological response (ACPR) [23]. Haemoglobin concentrations during follow-up were expressed as a percentage of the enrolment concentration. To quantify the effect of treatment on gametocyte densities, we determined the area under the curve (AUC) of *Pfs25* QT-NASBA gametocyte density versus time [24], [25]. This measure incorporates both the magnitude and the duration of gametocyte carriage and was described by Méndez *et al.* [25]. The AUC from days 0–42 was calculated as: AUC = [(3−0)×(g_0_+g_3_)/2+(7−3)×(g_3_+g_7_)/2+(14−7)×(g_7_+g_14_)/2+(28−14)×(g_14_+g_28_)/2+(42−28)×(g_28_+g_42_)/2]/42; where g_d_ represents *Pfs25* QT-NASBA gametocyte density on day d. Gametocyte negative samples were included as zeroes. The measure was scaled by 42 so that it represents AUC per day and this was transformed by log10. Microscopic and QT-NASBA parasite densities were analysed after log10-transformation. Because we were interested in clearance of gametocytes of the original infection, slides from individuals in which PCR analysis defined a new infection were excluded from analyses on post-treatment gametocyte prevalence and density.

Proportions were compared using the chi-squared statistic for a 2-by-2 contingency table. Normally-distributed continuous variables were compared using the Student t-test. Variables that were not normally distributed were compared using the Wilcoxon rank-sum test. Multiple linear regression models were used in case of continuous variables to adjust for potential confounding factors such as asexual parasite and gametocyte density at enrolment. Multiple logistic regression models with Generalized Estimating Equations (GEE) were used to test the influence of treatment on dichotomous variables with multiple observations per participant, such as gametocyte prevalence during follow-up. Estimates were adjusted for potential confounding factors and a random effect was included in the models to allow for correlations within individuals. Regression coefficients (β) were calculated for continuous dependent variables and odds ratios (OR) for dichotomous dependent variables, both with 95% confidence intervals (95% CI). Statistical analyses were performed using SPSS for Windows 12.0 (SPSS Inc., Chicago, USA) and Stata 8.0 (Stata Corporation, Texas, USA).

## Results

### Recruitment and Participant Flow

**Figure 1:**
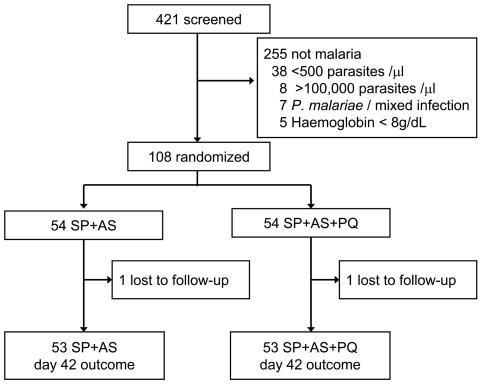
Profile of the study

A total of 108 children were randomised over the two treatment arms (figure 1). This number exceeds the original sample size (100) because of a high number of patients appearing on the last day of enrolment. Two children (1.9%) were lost for evaluation during the 42-day follow-up period, one in each treatment arm. Parasite density, gametocyte prevalence, haemoglobin concentrations, fever prevalence and the proportion of G6PD-deficient children at enrolment were not different between the treatment regimens (table 1). Adequate clinical and parasitological response (ACPR) on day 42 after treatment was observed in 71.7% (38/53) of the children treated with SP+AS and in 67.9% (36/53) of those treated with SP+AS+PQ (χ^2^ = 1.70; p = 0.64)(table 2).

### Outcomes and Estimation: Gametocyte carriage after treatment

**Table 1:** Characteristics of the study population at enrolment

	*SP+AS*	*SP+AS+PQ*
N	54	54
Age, median (IQR)	5 (3–8)	5.5 (3–10)
Sex, % male (n/N)	46.3 (25/54)	55.6 (30/54)
Temperature, % fever (>37.5°C) (n/N)	30.2 (16/53)	35.8 (19/53)
Asexual parasite density GM/µL (IQR)	4,759 (959–22,248)	7,379 (2,334–25,304)
Microscopic gametocyte prevalence, % (n/N)	26.4 (14/53)	18.9 (10/53)
*Pfs25* QT-NASBA gametocyte prevalence, % (n/N)	88.2 (45/51)	90.6 (48/53)
*Pfs25* QT-NASBA gametocyte density, GM/µL (IQR) *	28.8 (6.9–109.9)	17.5 (1.1–76.9)
G6PD, % (n/N)		
B	68.5 (37/54)	75.5 (40/53)
A	25.9 (14/54)	17.0 (9/53)
A-	5.6 (3/54)	7.5 (4/53)
Haemoglobin concentration, g/dL, median (IQR)	10.5 (9.4–11.8)	10.8 (9.8–11.8)

IQR = interquartile range; GM = geometric mean; G6PD: B = no G202A mutation; A = heterozygotes, single G202A mutation; A- = homozygote or hemizygote (males), only G202A mutations. ^*^For gametocyte carriers only.

**Table 2:** Treatment outcome for the different treatment regimens on day 14, 28 and 42.

	*SP+AS*	*SP+AS+PQ*
Number evaluated	53	53
Day 14 Treatment outcome, % (n)		
ACPR	98.1 (52)	100.0 (53)
ETF	0 (0)	0
LTF	0 (0)	0
Re-infection	1.9 (1)	0
Indeterminate	0 (0)	0
Day 28 Treatment outcome, % (n)		
ACPR	77.4 (41)	83.0 (44)
ETF	0 (0)	0 (0)
LTF	3.8 (2)	5.7 (3)
Re-infection	15.1 (8)	9.4 (5)
Indeterminate	3.8 (2)	1.9 (1)
Day 42 Treatment outcome, % (n)		
ACPR	71.7 (38)	67.9 (36)
ETF	0 (0)	0 (0)
LTF	3.8 (2)	9.4 (5)
Re-infection	17.0 (9)	13.2 (7)
Indeterminate	7.5 (4)	9.4 (5)

ACPR = adequate clinical and parasitological response, ETF = early treatment failure, LTF = late treatment failure. Indeterminate = indeterminate due to PCR-failure (n = 1) or missing filter paper DNA samples (n = 8).

**Figure 2:**
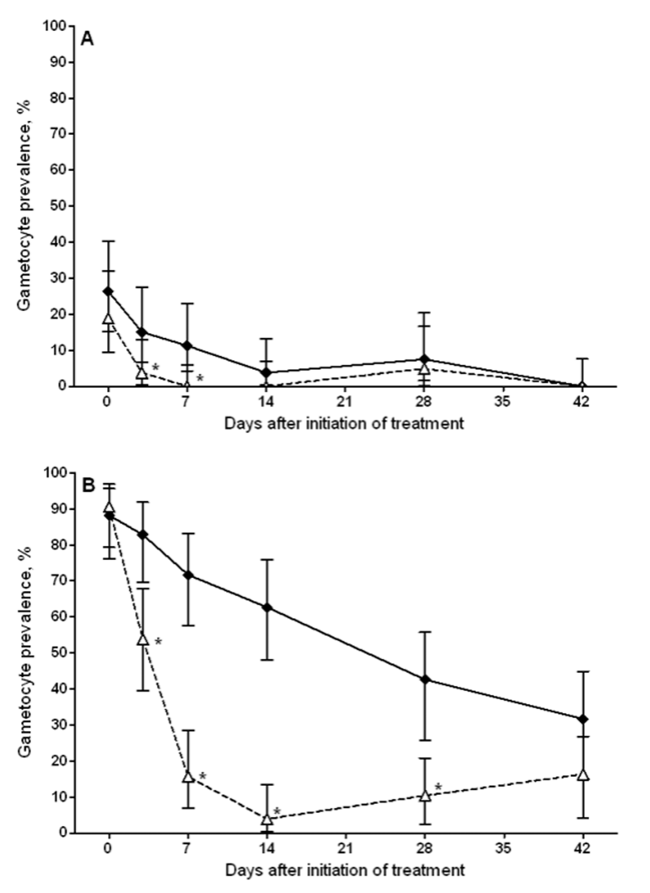
Gametocyte prevalence by microscopy (A) and *Pfs25* QT-NASBA (B). Gametocyte prevalence for SP+AS (closed diamonds, solid line) and SP+AS+PQ (open triangles, broken lines) treated children. Bars indicate the 95% confidence intervals around the proportions. * indicates a statistically significant difference between the two treatment arms.

Microscopic gametocyte prevalence at enrolment was 26.4% (14/53) for children treated with SP+AS and 18.9% (10/53) for children treated with SP+AS+PQ (table 1). Microscopic gametocyte prevalence decreased after treatment in both treatment arms (figure 2A) and was significantly lower in individuals treated with SP+AS+PQ compared to those treated with SP+AS when the entire period of follow-up was considered, after adjustment for enrolment gametocyte prevalence (GEE: OR = 0.17 (95% CI = 0.049–0.57), p = 0.004). No microscopic gametocyte carriage was seen on day 7 and 14 after treatment with SP+AS+PQ.

Enrolment gametocyte prevalence defined by *Pfs25* QT-NASBA was 88.2% (45/51) for SP+AS treated individuals compared to 90.6% (48/53) for SP+AS+PQ treated children (table 1). Predictably, children with microscopically confirmed gametocytes at enrolment had a significantly higher median *Pfs25* QT-NASBA gametocyte density (46.6 gametocytes/µL; IQR 8.9–346.5) compared to those gametocyte-free by microscopy (7.5 gametocytes/µL; IQR 3.1–67.5)(Wilcoxon Rank-Sum test z = −2,01, p = 0.04).

*Pfs25* QT-NASBA gametocyte density was not initially defined as outcome measure, but densities were compared between the treatment arms post-hoc. At the time of enrolment *Pfs25* QT-NASBA gametocyte density was 28.8 gametocytes/µL (IQR 6.9–109.9 gametocytes/µL) for SP+AS treated children compared to 17.5 gametocytes/µL (IQR 1.1–76.9) for SP+AS+PQ treated children (table 1). During follow-up *Pfs25* QT-NASBA gametocyte prevalence decreased for both SP+AS and SP+AS+PQ treated children (figure 2B and table 3). After adjustment for enrolment *Pfs25* QT-NASBA gametocyte density, *Pfs25* QT-NASBA gametocyte prevalence was lower in individuals treated with SP+AS+PQ during the entire period of follow-up (GEE: OR = 0.27 (95% CI = 0.18–0.40), p<0.001). The *Pfs25* QT-NASBA gametocyte density in gametocyte positive samples was consistently lower for SP+AS+PQ treated children during follow-up (table 3). The average duration of gametocyte carriage was summarised in the area under the curve of *Pfs25* QT-NASBA gametocyte density versus time (table 4). The area under the curve was significantly lower for SP+AS+PQ treated children (t-test t = 3.28, p <0.001), as were the number of sampling times when gametocytes were detected (GEE: OR = 0.27 (95% CI = 0.18–0.40), p<0.001) and the geometric mean gametocyte density in gametocyte positive samples (GEE: β = −0.40 (95% CI = −0.74−−0.07), p = 0.02).

**Table 3:** *Pfs25* QT-NASBA gametocyte prevalence and density after treatment with SP+AS and SP+AS+PQ.

	SP+AS		SP+AS+PQ	
	Gametocyte prevalence, % (n/N)	Gametocyte density/µL, GM (IQR)	Gametocyte prevalence, % (n/N)	Gametocyte density/µL, GM (IQR)
Day 0	88.2% (45/51)	28.8 (6.9–109.9)	90.6% (48/53)	17.5 (1.1–76.9)
Day 3	80.8% (42/52)	25.5 (4.2–132.9)	53.8% (28/52)	2.9 (0.6–36.6)
Day 7	71.7% (38/53)	8.0 (1.8–59.1)	15.7% (8/51)	0.3 (0.02–2.4)
Day 14	62.7% (32/51)	4.5 (1.8–37.1)	3.9% (2/51)	0.07&0.09
Day 28	41.9% (18/43)	10.9 (1.0–70.0)	10.4% (4/46)	3.8 (0.2–2.4)
Day 42	28.2% (11/39)	17.4 (1.2–191.4)	16.3% (5/40)	7.9 (0.5–54.3)

GM = geometric mean *Pfs25* QT-NASBA gametocyte density per microlitre for gametocyte carriers only; IQR = interquartile range

**Table 4:** Effect of treatment on gametocyte carriage during follow-up

	*SP+AS*	*SP+AS+PQ*	*p-value*
Mean AUC of gametocyte density/µL versus time, (IQR)	11.1 (2.2–53.8)	1.5 (0.3–8.8)	<0.001*
Number of sampling times when gametocytes were detected, % (n/N)	64.4 (186/289)	32.4 (95/293)	<0.001†
GM gametocyte density/µL on days when gametocytes were detected, (IQR)	15.8 (4.1–85.8)	5.8 (0.8–55.1)	0.02†

AUC = area under the curve; GM = geometric mean; IQR = interquartile range

* Adjusted for gametocyte density at enrolment; ^† ^adjusted for correlations between observations from the same individual

Most treatment failures or re-infections appeared after day 14 (table 2). It is therefore appropriate to focus on the first two weeks after treatment to determine the effect of treatment in the absence of treatment failure or re-infection. On day 14 after SP+AS treatment, 32 (62.7%) individuals were gametocyte positive with a mean gametocyte density of 4.5 gametocytes per µL (IQR 1.8–37.1)(table 3). Only two individuals (3.9%) were positive by *Pfs25* QT-NASBA on day 14 after treatment with SP+AS+PQ with low gametocyte densities of 0.067 and 0.094 gametocytes per µL.

### Haemoglobin concentrations after treatment

**Figure 3:**
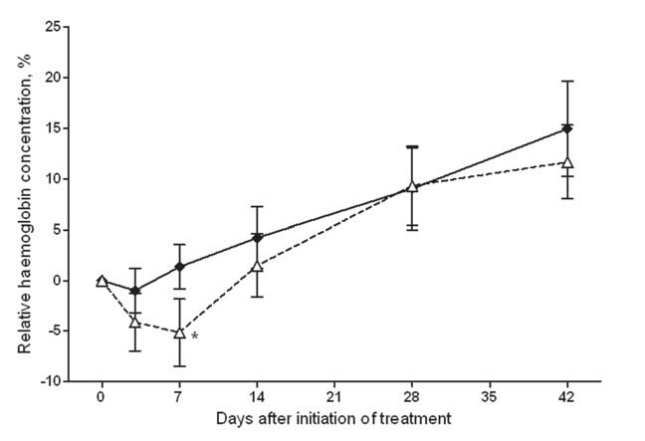
Haemoglobin concentration following treatment. Concentrations are expressed relative to that at enrolment for SP+AS (closed diamonds, solid line) and SP+AS+PQ (open triangles, broken lines). Bars indicate the 95% confidence intervals around the proportions. * indicates a statistically significant difference between the two treatment arms.

To address concerns about haemolysis associated with PQ use in G6PD deficient individuals, Hb concentration was assessed at enrolment and during follow-up. Median Hb at enrolment was 10.5 g/dL (IQR 9.4–11.8) for SP+AS treated children and 10.8 g/dL (9.8–11.8) for SP+AS+PQ treated children (table 1). While Hb relative to enrolment concentration gradually increased in the weeks after treatment with SP+AS, it first decreased for SP+AS+PQ treatment and remained lower up to day 14 (figure 3). The relative decrease was most pronounced on day 7 after SP+AS+PQ treatment when Hb concentration was 5.2% lower (95% CI −8.4–−1.8) than on enrolment (paired t-test: t = 2.86; p = 0.006). When only children with the G6PD B variant were considered, the relative decrease in Hb concentration on day 7 after treatment with SP+AS+PQ was no longer statistically significant (paired t-test: t = 1.57; p = 0.12).

**Figure 4:**
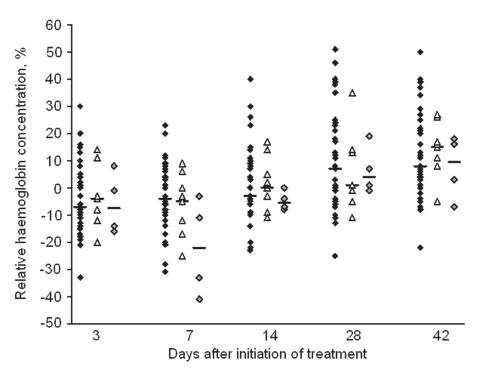
Relative haemoglobin concentration after treatment with SP+AS+PQ for different G6PD genotypes. Haemoglobin concentration relative to enrolment for children without the G202A mutation (G6PD genotype B; black diamonds, n = 39), heterozygotes (G6PD genotype A; white triangles, n = 9) and homozygotes or hemizygotes (G6PD genotype A-: grey diamonds, n = 4). Each individual measurement is shown; lines indicate the median value.

The reduction in Hb shortly after SP+AS+PQ treatment was most pronounced in children with the G6PD A- variant (figure 4) although numbers were too small to allow statistical comparisons. None of the children developed clinical symptoms related to anaemia or an Hb below 5g/dL. The Hb concentrations on day 28 and day 42 after treatment were equal to or greater than that at enrolment for all G6PD categories (figure 4). Eight children experienced ≥20% reduction in Hb concentration on day 7 relative to that at enrolment, compared to none in the SP+AS treatment arm. The range of Hb concentration in these eight individuals was 5.4–10.4 g/dL on day 7 after SP+AS+PQ treatment. Two of the children with ≥20% reduction in Hb concentration on day 7 had the G6PD A- variant (25%), one had the G6PD A variant (12.5%) and the other five had G6PD B variant (62.5%). None of the children reported symptoms suggestive of anaemia or allergic drug reactions during follow-up.

## Discussion

### Interpretation

This study shows that a single dose of primaquine (PQ) is of significant additive value in clearing gametocytes in an area of high malaria endemicity in Tanzania. Only 3.9% of children treated with SP+AS+PQ had gametocytes on day 14 after successful treatment compared to 62.7% for SP+AS treated children. Gametocytes persisting on day 14 after SP+AS+PQ treatment circulated at densities well below the theoretical threshold for mosquito infection.

Gametocyte carriage after treatment with SP+AS persisted for more than one month and more than two-thirds of the treated individuals harboured gametocytes on day 14. These data confirm previous findings [26]. The addition of a single dose of PQ to this regime significantly reduced gametocyte carriage. When microscopy was used as tool to detect gametocytes, no gametocytes were observed seven days after the initiation of treatment with SP+AS+PQ. On day 28 after treatment, gametocytes were detected in only one individual. Submicroscopic gametocyte prevalence decreased to 3.9% (2/51) on day 14 after initiation of treatment but increased at subsequent follow up time points. This increase is most likely due to recrudescence of infection or new infections that were undetected by microscopy. Treatment failure rates were similar to those reported in recent drug efficacy studies in east Africa [3], [4], [27] and re-infection rates were high in this area of intense malaria transmission. As a consequence, 28.3% of the SP+AS treated children and 32.1% of the SP+AS+PQ treated children experienced microscopically confirmed treatment failure or re-infection during follow-up. True treatment failure and re-infection rates may be higher as not all asexual parasites will be detected by microscopy. A high persistence of submicroscopic asexual parasites after apparently successful drug treatment has been described [28], as well as the acquisition of new sub-patent infections [29]. Gametocytes that newly developed from new or persisting asexual infections are therefore likely to be responsible for the increase in gametocyte prevalence after day 14 that has been reported previously [5].

Focusing on the first two weeks after initiation of treatment, PQ seems to decrease the number of gametocytes rapidly to a level where onward transmission may be arrested completely. Although analyses on gametocyte densities should be considered as post-hoc analyses and we did not directly determine post-treatment malaria transmission, it is clear that the transmission potential is reduced in children treated with SP+AS+PQ. Gametocyte prevalence and density were lower in children treated with SP+AS+PQ and gametocytes persisting on day 14 after treatment circulated at densities below 0.1 gametocyte/µL. When assuming an average mosquito blood meal size of 2–3 µL [30], these concentrations are unlikely to result in mosquito infection, although infectiousness of very low gametocyte densities can not be excluded [31].

### Generalisability

The rationale for using PQ in *P. falciparum* infections is to reduce post-treatment infectivity (as routinely practiced in several countries in Asia and the Americas [1]) and as a quarantine treatment to reduce the spread of (drug resistant) parasites. The aim of the current study was not to determine if PQ should be routinely added to current ACT regimens. The objective was to explore if PQ is a valuable component in mass drug administration studies that aim to reduce malaria transmission. Mass drug administration (MDA) studies are typically conducted in areas of low malaria transmission intensity and also include asymptomatic parasite carriers. The likelihood of re-infection is lower in these areas and the effect of PQ can be expected to be larger because enrolment gametocyte densities are lower.

In MDA studies, drugs are administered to asymptomatic individuals, raising safety concerns over the haemolytic effect of PQ on individuals with G6PD mutations. Due to the potential protective effect of G6PD-deficiency from malaria, G6PD deficiency is probably selected in malaria endemic regions, in a similar manner as for other haemoglobinopathies [32]. In our population, 6.5% (7/107) of the children had the A- variant of G6PD deficiency. We observed that the addition of PQ resulted in a statistically significant but transient reduction in haemoglobin levels, as was previously reported for PQ administered at a curative dose (0.5 mg/kg, 14 days) to individuals with the African Variant (A-) of G6PD-deficiency [10]. The observed reduction in Hb concentration indicates a small but genuine risk of PQ use in individuals who are anaemic prior to drug administration. The risk to the individual patient has to be weighted against the potential benefit of a reduced malaria transmission. In the current study, we excluded individuals with an Hb<8 g/dL. The risk of anaemia is likely to be increased in individuals with lower pre-treatment Hb concentrations, which is important since Hb is not routinely determined prior to treatment. In case of mass-administration of SP+AS+PQ, anaemic individuals should preferably be identified prior to treatment and excluded from PQ treatment. This can be done by Hemocue® which allows a rapid and reliable assessment of anaemia in the field [33]. Areas of low and seasonal malaria transmission are those that are most likely to benefit from MDA. In these areas, the prevalence of severe anaemia prior to the malaria season is likely to be low [34]. In these circumstances we consider the addition of a single dose of PQ to SP+AS to be a safe approach to reduce post-treatment malaria transmission.

### Overall evidence

This is the first study that determines the effect of PQ on submicroscopic gametocyte densities and our findings are in line with previous studies that where PQ efficiently cleared microscopic gametocyte concentrations [10], [11], [35]–[37]. The addition of a single dose of PQ had no beneficial influence on the clearance of asexual parasites, as was described previously [36], [38].

## Supporting Information

Checklist S1 CONSORT Checklist
(0.05 MB DOC)

Protocol S1 Trial Protocol
(0.21 MB DOC)

## References

[1] World Health Organization (2006). Guidelines for the treatment of malaria.. WHO/HTM/MAL/2006..

[2] von Seidlein L, Greenwood BM (2003). Mass administrations of antimalarial drugs.. Trends Parasitol.

[3] Adjuik M, Babiker A, Garner P, Olliaro P, Taylor W, White N (2004). Artesunate combinations for treatment of malaria: meta-analysis.. Lancet.

[4] Bousema JT, Schneider P, Gouagna LC, Drakeley CJ, Tostmann A (2006). Moderate Effect of Artemisinin-Based Combination Therapy on Transmission of Plasmodium falciparum.. J Infect Dis.

[5] Drakeley CJ, Jawara M, Targett GAT, Walraven G, Obisike U (2004). Addition of artesunate to chloroquine for treatment of Plasmodium falciparum malaria in Gambian children causes a significant but short-lived reduction in infectiousness for mosquitoes.. Trop Med Int Health.

[6] Sutherland CJ, Ord R, Dunyo S, Jawara M, Drakeley CJ (2005). Reduction of Malaria Transmission to Anopheles Mosquitoes with a Six-Dose Regimen of Co-Artemether.. PLoS Med.

[7] Targett G, Drakeley C, Jawara M, von Seidlein L, Coleman R (2001). Artesunate reduces but does not prevent posttreatment transmission of Plasmodium falciparum to Anopheles gambiae.. J Infect Dis.

[8] Nosten F, van Vugt M, Price R, Luxemburger C, Thway KL (2000). Effects of artesunate-mefloquine combination on incidence of Plasmodium falciparum malaria and mefloquine resistance in western Thailand: a prospective study.. Lancet.

[9] Butcher GA (1997). Antimalarial drugs and the mosquito transmission of Plasmodium.. Int J Parasitol.

[10] Taylor WR, White NJ (2004). Antimalarial drug toxicity: a review.. Drug Saf.

[11] Pukrittayakamee S, Chotivanich K, Chantra A, Clemens R, Looareesuwan S (2004). Activities of artesunate and primaquine against asexual- and sexual-stage parasites in falciparum malaria.. Antimicrob Agents Chemother.

[12] Weerasinghe KL, Galappaththy G, Fernando WP, Wickremasinghe DR, Faizal HM, Wickremasinghe AR (2002). A safety and efficacy trial of artesunate, sulphadoxine-pyrimethamine and primaquine in P falciparum malaria.. Ceylon Med J.

[13] Lusingu JP, Vestergaard LS, Mmbando BP, Drakeley CJ, Jones C, Akida J, Savaeli ZX, Kitua AY, Lemnge MM, Theander TG (2004). Malaria morbidity and immunity among residents of villages with different Plasmodium falciparum transmission intensity in North-Eastern Tanzania.. Malar J.

[14] Schneider P, Schoone G, Schallig H, Verhage D, Telgt D (2004). Quantification of Plasmodium falciparum gametocytes in differential stages of development by quantitative nucleic acid sequence-based amplification.. Mol Biochem Parasitol.

[15] Schneider P, Wolters L, Schoone G, Schallig H, Sillekens P (2005). Real-time nucleic acid sequence-based amplification is more convenient than real-time PCR for quantification of Plasmodium falciparum.. J Clin Microbiol.

[16] Boom R, Sol CJ, Salimans MM, Jansen CL, Wertheim-van Dillen PM (1990). Rapid and simple method for purification of nucleic acids.. J Clin Microbiol.

[17] Ponnudurai T, Lensen AH, Leeuwenberg AD, Meuwissen JH (1982). Cultivation of fertile Plasmodium falciparum gametocytes in semi-automated systems. 1. Static cultures.. Trans R Soc Trop Med Hyg.

[18] Wooden J, Kyes S, Sibley CH (1993). PCR and strain identification in Plasmodium falciparum.. Parasitol Today.

[19] Pearce RJ, Drakeley C, Chandramohan D, Mosha F, Roper C (2003). Molecular determination of point mutation haplotypes in the dihydrofolate reductase and dihydropteroate synthase of Plasmodium falciparum in three districts of northern Tanzania.. Antimicrob Agents Chemother.

[20] Snounou G, Zhu X, Siripoon N, Jarra W, Thaithong S (1999). Biased distribution of msp1 and msp2 allelic variants in Plasmodium falciparum populations in Thailand.. Trans R Soc Trop Med Hyg.

[21] Cattamanchi A, Kyabayinze D, Hubbard A, Rosenthal PJ, Dorsey G (2003). Distinguishing recrudescence from reinfection in a longitudinal antimalarial drug efficacy study: comparison of results based on genotyping of msp-1, msp-2, and glurp.. Am J Trop Med Hyg.

[22] Enevold A, Vestergaard LS, Lusingu J, Drakeley CJ, Lemnge MM (2005). Rapid screening for glucose-6-phosphate dehydrogenase deficiency and haemoglobin polymorphisms in Africa by a simple high-throughput SSOP-ELISA method.. Malar J.

[23] World Health Organization (2002). Monitoring Antimalarial Drug Resistance.. WHO/CDS/CSR/EPH/2002..

[24] Dunyo S, Milligan P, Edwards T, Sutherland C, Targett G (2006). Gametocytaemia after drug treatment of asymptomatic Plasmodium falciparum.. PloS Clinical Trials.

[25] Mendez F, Munoz A, Plowe CV (2006). Use of area under the curve to characterize transmission potential after antimalarial treatment.. Am J Trop Med Hyg.

[26] Schneider P, Bousema T, Omar S, Gouagna L, Sawa P (2006). (Sub)microscopic Plasmodium falciparum gametocytaemia in Kenyan children after treatment with sulphadoxine-pyrimethamine monotherapy or in combination with artesunate.. Int J Parasitol.

[27] Dorsey G, Njama D, Kamya MR, Cattamanchi A, Kyabayinze D (2002). Sulfadoxine/pyrimethamine alone or with amodiaquine or artesunate for treatment of uncomplicated malaria: a longitudinal randomised trial.. Lancet.

[28] Babiker A, Abdel-Muhsin AA, Ranford-Cartwright LC, Satti G, Walliker D (1998). Characteristics of Plasmodium falciparum parasites that survive the lengthy dry season in eastern Sudan where malaria transmission is markedly seasonal.. Am J Trop Med Hyg.

[29] Ofosu-Okyere A, Mackinnon MJ, Sowa MP, Koram KA, Nkrumah F (2001). Novel Plasmodium falciparum clones and rising clone multiplicities are associated with the increase in malaria morbidity in Ghanaian children during the transition into the high transmission season.. Parasitology.

[30] Menge DM, Guda T, Zhong D, Pai A, Zhou G (2005). Fitness consequences of Anopheles gambiae population hybridization.. Malar J.

[31] Schneider P, Bousema JT, Gouagna LC, Otieno S, van de Vegte-Bolmer M (2007). Submicroscopic Plasmodium falciparum gametocyte densities frequently result in mosquito infection.. Am J Trop Med Hyg.

[32] Kwiatkowski DP (2005). How malaria has affected the human genome and what human genetics can teach us about malaria.. Am J Hum Genet.

[33] Schellenberg D, Schellenberg JR, Mushi A, Savigny D, Mgalula L (2003). The silent burden of anaemia in Tanzanian children: a community-based study.. Bull World Health Organ.

[34] Shekalaghe SA, Bousema JT, Kunei KK, Lushino P, Masokoto A (2007). Submicroscopic Plasmodium falciparum gametocyte carriage is common in an area of low and seasonal transmission in Tanzania.. Trop Med Int Health.

[35] Gogtay NJ, Kamtekar KD, Dalvi SS, Mehta SS, Chogle AR (2006). A randomized, parallel study of the safety and efficacy of 45 mg primaquine versus 75 mg bulaquine as gametocytocidal agents in adults with blood schizonticide-responsive uncomplicated falciparum malaria [ISCRTN50134587].. BMC Infect Dis.

[36] Lederman ER, Maguire JD, Sumawinata IW, Chand K, Elyazar I (2006). Combined chloroquine, sulfadoxine/pyrimethamine and primaquine against Plasmodium falciparum in Central Java, Indonesia.. Malar J.

[37] Chen PQ, Li GQ, Guo XB, He KR, Fu YX (1994). The infectivity of gametocytes of Plasmodium falciparum from patients treated with artemisinin.. Chin Med J.

[38] Suputtamongkol Y, Chindarat S, Silpasakorn S, Chaikachonpatd S, Lim K (2003). The efficacy of combined mefloquine-artesunate versus mefloquine-primaquine on subsequent development of Plasmodium falciparum gametocytemia.. Am J Trop Med Hyg.

