# Supplementary material for: Primaquine Clears Submicroscopic Plasmodium falciparum Gametocytes that Persist after Treatment with Sulphadoxine-Pyrimethamine and Artesunate
Source: PLoS One. 2007 Oct 10;2(10):e1023. doi: 10.1371/journal.pone.0001023 (PMC1995753; doi:10.1371/journal.pone.0001023)
Supplement: Protocol S1 — Trial Protocol (0.21 MB DOC) [file pone.0001023.s002.doc]

TITLE:

**The gametocytocidal activity of sulphadoxine-yrimethamine *plus* artesunate followed by a single dose of primaquine.**

**Principal Investigator**

Seif Shekalaghe, MD, MPH – principal investigator, PhD-candidate

Dr. Frank Mosha, MD, PhD

# Collaborators

Dr. Chris Drakeley, PhD

Dr. Roly Gosling, MD

Dr. Michael Alifrangis, PhD

Prof. Dr. Robert Sauerwein, MD, PhD

Teun Bousema, MSc

**Funding source**

PRIOR, The Netherlands

### Summary

Gametocytocidal drugs can play an important role in malaria control. A transmission reducing intervention is planned in a low transmission area in Lower Moshi. The current study proposal is a pilot study to determine the efficacy of a gametocytocidal drug combination that consists of the extensively tested combination of sulphadoxine-pyrimethamine (SP) plus artesunate (AS), followed by a single dose of primaquine (PQ). This combination has previously been used and has been shown to be safe and efficient in clearing microscopic gametocytes. In this study we propose to confirm the safety in a Tanzanian population and to determine the added value of a single dose of PQ on the clearance of (sub-microscopic) gametocytes compared to SP+AS alone.

**BACKGROUND:**

Malaria is one of the leading infectious diseases in the world; it is endemic throughout tropical and subtropical regions. Yearly, over 500 million clinical malaria episodes need anti-malaria treatment throughout the world. The disease has tremendous impact on the quality of life and reduction of economic growth; worldwide malaria causes an economic loss of US$ 12 billion annually and it contributes significantly to the ongoing poverty in endemic areas. In Tanzania, malaria remains the largest single cause of mortality and morbidity, accounting for 34% of deaths amongst under 5 years, and 23% of deaths amongst over 5 years olds (Ministry of Health Tanzania, 2000).

Among the four species of malaria parasites, *Plasmodium falciparum* is the most dangerous form for humans and responsible for large number of human casualties, mainly young children in Africa. The clinical disease is caused by asexual forms of *P. falciparum* in the blood of the infected persons. A small proportion of these differentiate into stages called gametocytes. Gametocytes, the sexual stage malaria parasites, are responsible for transmission of malaria from man to mosquito during feeding of *Anopheles* mosquitoes. Drugs that reduce gametocytaemia are expected to have a beneficial effect on malaria transmission^1-3^. This study aims at finding the best combination for preventing malaria transmission by comparing two antimalarial drug combinations. This drug combination could subsequently be used in a large scale transmission reducing intervention in a low transmission area.

### DRUG-COMBINATIONS FOR REDUCING MALARIA TRANSMISSION

Antimalarial drugs that will be used for transmission reducing interventions need to be safe, effective in clearing asexual parasites and have a profound effect on gametocytes. Artemisinin based combination therapy fulfils all these criteria. The combination of sulphadoxine-pyrimethamine (SP) and a three dose regimen of artesunate (AS) has been extensively tested in Tanzania, Uganda and Kenya^2-5^, is safe and efficacious in clearing asexual parasites and reduces gametocytaemia^2,3^. In pregnant women, no negative effects of AS were reported.^6-8^ SP + AS is, however, not capable of clearing all gametocytes and is therefore unable to fully prevent malaria transmission. ^1,3^ Although primaquine (PQ) is not suitable for clearing *P. falciparum* asexual parasites as monotherapy, it is the only drug that has been shown to actively clear *P. falciparum* gametocytes^8,9^. PQ has previously been used in the lower Moshi area without any serious side effects reported. In addition, it was recently used in combination with SP and AS and found to be safe and highly efficacious in clearing all parasite stages^10^ . However, there is concern for negative haemolytic side effects that have been reported for the full dose regimen in individuals who are G6PD deficient^8^. Intravascular haemolysis is most severe in high doses and in people with the Mediterranean and Asian variants of G6PD deficiency. It is relatively mild in those with African variants.^11^ As a consequence of possible haemolysis, a curative dose of PQ is contraindicated in pregnancy and in children under 4 years of age because of the risk of haemolysis^12^. Haemolytic side effects are generally self-limiting and, more importantly, are observed in individuals receiving the full dose PQ treatment (0.5 mg/kg, 14 days). A study in African-Americans showed that anaemia (9-10g/dl) was seen after 4-9 days of primaquine use in 4% of the individuals (reviewed in ^8^). Despite these limitations of PQ as full-dose treatment, PQ can also be used to clear gametocytes after clearing asexual parasites with a different drug or drug-combination. This gametocytocidal treatment requires a single dose of PQ only and is mentioned by the World Health Organization (WHO) as beneficial in areas of low malaria transmission. PQ has a short half-life of 5-6 hours^11^ and the single dose of 0.75mg/kg (adults 45mg) is considered too low to cause haemolytic effects. According to WHO standards, testing for G6PD deficiency is therefore not required^12^.

In the current study, we want to determine the added value of a single dose of primaquine to the standard treatment of SP + AS. The principle outcomes will be safety and gametocyte prevalence by sensitive molecular detection methods.

**JUSTIFICATION**

It is explicitly not the study aim to find a drug combination that can replace first line antimalarial treatment. This study can be considered as a pilot study for a future mass drug administration study (MDA). An MDA with a gametocytocidal drug combination can be a useful tool for malaria control in low transmission areas and will provide information that is highly relevant for planning other transmission reducing interventions, such as malaria transmission blocking vaccines. Before an MDA study can be planned, the safety and efficacy of the used gametocytocidal drug combination has to be tested. Although the drug combination we propose has been used before^10^, and is considered as a safe approach by WHO^12^, we therefore propose a small scale study to confirm safety and efficacy in a Tanzanian population. Individuals will be followed up for 42 days to allow very detailed information on gametocyte clearance.

**OBJECTIVES**

- To confirm the safety of sulphadoxine-pyrimethamine (SP, 1 day) + artesunate (AS, 3 days) followed by primaquine (PQ, 1 day)
- To determine the added value of a single dose of primaquine for the clearance of (submicroscopic levels of) gametocytes.
- To transfer knowledge, and training for use of the G6PD PCR and QT-NASBA technology to the Biotechnology Laboratory at KCMC, Moshi.

# DESIGN AND METHODOLOGY

**Study site (Geographical)**

The future MDA study is planned in lower Moshi. This area is characterised by a very low transmission intensity, making it very suitable for transmission reducing interventions. Because of its low transmission intensity it is, however, not ideal for a small scale clinical study comparing antimalarial drug combinations in uncomplicated malaria patients. We therefore propose to conduct this study in a clinic in Mnyuzi in the vicinity of Korogwe. Transmission intensity is higher in this area and our study will not be interfering with any ongoing malaria programmes. Mnyuzi is only selected for this pilot study and we have absolutely no intentions to conduct studies reducing malaria transmission in this area. We propose to exclude all individuals who are eligible for other currently running malaria programmes such as the IPTi project.

## Study population

Individuals aged 3-15 years and above visiting the clinic hospitals with signs of uncomplicated malaria and residing in the research area (5 km around the clinic) will be eligible for the study. Because safety is one of the primary interests, we choose to exclude the younger children and exclude those with moderate or severe anemia (<8g/dl).

Inclusion criteria

- Age 3-15 years
- Residents of research area (5 km around the clinic)
- Willingness to come for complete scheduled follow-up.
- Uncomplicated malaria with *P. falciparum* mono-infection
- Parasitaemia of 500-100 000 parasites/ul
- Temperature > 37.5 and < 39.5°C, or history of fever in the previous 24 hours.
- No history of adverse reactions to SP or PQ treatment
- Understanding of the procedures of the study by parent or guardian and willing to participate by signing informed consent forms.

Exclusion criteria

- General signs of severe malaria or haemoglobin count < 8g/dl.
- Presence of disease other than malaria causing febrile conditions
- Unwilling to participate and sign informed consent forms.
- Eligibility for (or participation in) other malaria projects in the Korogwe area, notably the IPTi study.

**SAMPLE SIZE CALCULATION**

Sample size calculations are based on GEE analyses with 5 time points at

which gametocyte carriage is assessed. The estimated number of

individuals in the study is based on the assumption of a maximum correlation

between observations of the same individual of 0.30, similar population

sizes for both treatment arms, enrolment (submicroscopic) gametocyte

prevalence of 80-90% and an average (submicroscopic) gametocyte prevalence

after SP+AS treatment of 58%^3^. When a minimum reduction in gametocyte carriage of 50% is considered as relevant, the minimum number of individuals per treatment arm is 41. Based on these calculations, we will include 50 individuals per treatment arm.

## PROCEDURES, FIELDWORK AND LABORATORY ANALYSES

## Enrolment and blood sampling

All those presenting at the outpatient clinic will be informed about the study. Finger prick blood sample will be taken from everyone presenting with symptoms of uncomplicated malaria. Parasite detection will be based on microscopy. Blood smears will be Giemsa-stained and asexual parasites and gametocytes counted against 100 high power fields.

Those who are blood slide positive and that meets all inclusion criteria will get detailed explanation about the purpose of the study and procedure and will be enrolled for study, once they (or their parents/guardians) have understood and signed an informed consent. Name, address, age, weight, and clinical symptoms will be recorded on a patient card. Individuals not included in the study will be seen by clinician and be treated accordingly or will receive treatment for malaria according to national guidelines. From enrolled individuals, an additional finger prick blood sample will be taken to measure haemoglobin using Haemocue and to extract parasite nucleotides for QT-NASBA parasite quantification.

# Treatment

Participants will be randomised to treatment with:

1. sulfadoxine-pyrimethamine (SP) (500 mg S; 25 mg P/20 kg body weight, day0) and artesunate (4 mg/kg, day 0, 1, 2) followed by a placebo (day 2)
2. SP (500 mg S; 25 mg P/20 kg body weight, day 0) and AS (4 mg/kg, day 0, 1, 2) followed by primaquine (PQ)(0.75mg/kg, day 2).

Randomisation will take place by the study physician who has envelopes for each study participant giving either i) SP+AS or ii) SP+AS+PQ.

PQ (and placebo) will be given with food to minimize the chance of (mild) gastro-intestinal side effects. Treatment is summarized in table 1. Participants will be screened for G6PD deficiency to monitor the possible effect of this status on side effects. It will however not influence randomization, since this is not indicated according to WHO standards^12^. A unique identification number (IDNR) will be assigned to all participants and written on the patient card. Patient cards of the study participants will be available only to the investigators directly involved with the child for clinical care and follow-up. All treatment will be given free of charge.

## Table 1. Treatment

|  |  | **Sulphadoxine-pyrimethamine** | **Artesunate** | **Primaquine** |
| --- | --- | --- | --- | --- |
|  |  | 500 mg S/20kg  25 mg P/20 kg | 4 mg/kg | 0.75mg/kg |
| Day 0 | Clinic | X | X |  |
| Day 1 | Clinic |  | X |  |
| Day 2 | Clinic |  | X | x |

## Follow up

Participants will be clinically examined on days 0, 3, 7, 14, 28, and 42. In addition, they are invited to come to the clinic at any day symptoms occur. On each follow-up day a blood slide will be taken and 50μl blood sample for QT-NASBA and haemoglobin measurement. Day 3 is an exception. This follow-up visit is only included to closely monitor possible haemolytic activity of PQ and therefore only Hb will be measured. The QT-NASBA samples will be processed at the KCMC and analysed in Nijmegen, The Netherlands. In case of treatment failure, participants will be treated with mefloquine (15 mg/kg on first day and 10 mg/kg on second day). Parameters determined during follow-up are summarised in the table below. Participants are free to withdraw from the study. This will have no influence on the care provided. Samples are taken from a single finger prick, as presented in table 2. Haemoglobin will be determined by HemoCue (HemoCue, Stockholm, Sweden), G6PD deficiency by PCR (collaboration with Michael Alifrangis and others).

# Table 2. Follow-up

|  | **Treatment** | **Blood smear** | **Haemoglobin** | **G6PD-deficiency** | **QT-NASBA** | **Genotyping** |
| --- | --- | --- | --- | --- | --- | --- |
| D0 | X | X | X | X | X | X |
| D1 | X |  |  |  |  |  |
| D2 | X |  |  |  |  |  |
| D3 |  |  | X |  |  |  |
| D7 |  | X | X |  | X | X^1^ |
| D14 |  | X | X |  | X | X^1^ |
| D28 |  | X | X |  | X | X^1^ |
| D42 |  | X | X |  | X | X^1^ |

All samples after enrolment are taken from a single finger prick blood sample.

^1^ only in case of treatment failure, a filter paper blood sample will be taken for genotyping.

**Parasite detection by microscopy and QT-NASBA**

Blood smears are stained for 10 minutes with 10% Giemsa and screened for asexual parasites and gametocytes. Slides are declared negative if no parasites are observed in 100 microscopic fields; asexual parasites and gametocytes counted against 200 and 500 white blood cells, respectively. All slides are re-read for quality control. Parasite detection on the 50μl filter paper blood samples is done by QT-NASBA as described elsewhere ^13,14^.The 18s QT-NASBA detects asexual parasites and gametocytes at a detection limit of 10 parasites/ml; the Pfs25 QT-NASBA is gametocyte specific and has a detection limit of 20-100 gametocytes/ml. Parasite detection by QT-NASBA will be done on a NucliSens EasyQ analyser (bioMérieux, Boxtel, The Netherlands).

**Genotyping**

To distinguish between recrudescence and re-infection, a filter paper blood sample will be taken at enrolment and at the day of treatment failure.

DNA will be extracted using a Chelex-based method. Alleles of the polymorphic locus *msp2* are compared between pre- and post-treatment parasite isolates by PCR as previously described ^3^.

**Data management**

Data will be recorded in questionnaire/data sheet, which will be double locked in cupboard with keys in possession of study supervisor. Data will be double entered in created computer access database and computer data will be protected by password available to study supervisor and principle investigator. After comparison of double entered files for validation, data will be exported to SPSS for statistical analysis. Principal outcomes in the statistical analyses will be gametocyte carriage detected by QT-NASBA and side-effects and haemoglobin levels stratified by treatment and G6PD status.

**Reference List**

1. Targett,G. *et al.* Artesunate reduces but does not prevent posttreatment transmission of Plasmodium falciparum to Anopheles gambiae. *Journal of Infectious Diseases* **183**, 1254-1259 (2001).

2. Adjuik,M. *et al.* Artesunate combinations for treatment of malaria: meta-analysis. *Lancet* **363**, 9-17 (2004).

3. Bousema,J.T. *et al.* Moderate effect of artemisinin-based combination therapy on transmission of *Plasmodium falciparum*. *Journal of Infectious Diseases* **in press** (2006).

4. Kachur,S.P. *et al.* Adherence to antimalarial combination therapy with sulfadoxine-pyrimethamine and artesunate in rural Tanzania. *Am. J. Trop. Med. Hyg.* **71**, 715-722 (2004).

5. Obonyo,C.O. *et al.* Artesunate plus sulfadoxine-pyrimethamine for uncomplicated malaria in Kenyan children: a randomized, double-blind, placebo-controlled trial. *Trans. R. Soc. Trop. Med. Hyg.* **97**, 585-591 (2003).

6. McGready,R. *et al.* Artemisinin antimalarials in pregnancy: a prospective treatment study of 539 episodes of multidrug-resistant Plasmodium falciparum. *Clin. Infect. Dis.* **33**, 2009-2016 (2001).

7. Phillips-Howard,P.A. & Wood,D. The safety of antimalarial drugs in pregnancy. *Drug Saf* **14**, 131-145 (1996).

8. Taylor,W.R. & White,N.J. Antimalarial drug toxicity: a review. *Drug Saf* **27**, 25-61 (2004).

9. Pukrittayakamee,S. *et al.* Activities of artesunate and primaquine against asexual- and sexual-stage parasites in falciparum malaria. *Antimicrob Agents Chemother* **48**, 1329-1334 (2004).

10. Weerasinghe,K.L. *et al.* A safety and efficacy trial of artesunate, sulphadoxine-pyrimethamine and primaquine in P falciparum malaria. *Ceylon Med. J.* **47**, 83-85 (2002).

11. Warrel,D.A., Watkins,W.M. & Winstanley,P.A. Essential Malariology. Warrel,D.A. & Gilles,H.M. (eds.), pp. 268-312 (BookPower, London,2003).

12. World Health Organization. The use of antimalarial drugs. Report of an informal consultation. WHO/CDS/RBM/2001.33. 2001. World Health Organization, Geneva, Switzerland.

13. Schneider,P. *et al.* Quantification of Plasmodium falciparum gametocytes in differential stages of development by quantitative nucleic acid sequence-based amplification. *Mol. Biochem. Parasitol.* **137**, 35-41 (2004).

14. Schneider,P. *et al.* Real-time nucleic acid sequence-based amplification is more convenient than real-time PCR for quantification of Plasmodium falciparum. *J. Clin. Microbiol.* **43**, 402-405 (2005).

# APPENDIX A: BUDGET

# Staff

1 medical doctor (KCMC) --

2 lab technicians (PRIOR/KCMC) --

3 local health workers for follow-up 900

Expenses use of clinic 400

**Drugs**

- Sulph-pyrimeth (Roche, Switzerland) (100 d) 50

- Artesunate (Sanofi, France) (300 d) 240

- Primaquine (base) (50 d) 125

- Mefloquine (Roche, Switzerland) (25 d) 150

- Paracetamol (1000 d) 20

# Durables

- Motorbike for follow-up 800

- Ear thermometers (2) 240

- HemoCue photometer 400

# Consumables

- Transport 1000

- Living costs KCMC personnel --

- HemoCue cuvettes (700p) 400

- Lancets (5000 p) 400

- Slides (5000 p) 100

- Cotton wool (4 rolls) 6

- Giemsa powder (50 g) 75

- Immersion oil (500 ml) 100

- Gloves (20 boxes, non-powdered) 80

- caps for earthermometer (500) 75

- Patient cards (150 p) 100

- Stickers 10

- Stationary 25

**QT-NASBA / genotyping**

- G6PD reagents 200

- MSP2 genotyping reagents 285

- Filterpaper (500 p) 150

- Silica (200 g) 80

- L6 QT-NASBA solution (0.75 l) 80

- L2 QT-NASBA washing solution (1 l) 125

- Ethanol (5 l) 50

- Methanol (1 l) 10

- Aceton (1 l) 20

- Commercial kits for RNA amplification (10 p) 2500

- Filtertips 200 ul (8*96 p) 65

- Filtertips 1000 ul (10*60 p) 55

- Eppendorf tubes safelock (1000 p) 150

- Storage boxes (6 p) 30

Total: 9496 EURO

**INFORMED CONSENT EXPLANATION**

(To be read and questions answered in a language in which the volunteer is fluent).

**TITLE OF STUDY:** **The gametocytocidal activity of sulphadoxine-pyrimethamine *plus* artesunate followed by a single dose of primaquine.**

.

**INSTITUTIONS**: Kilimanjaro Christian Medical Centre (KCMC), Moshi, Tanzania; Joint Malaria Programme (JMP), Moshi, Tanzania; University Medical Centre Nijmegen (UMCN).

**PRINCIPAL INVESTIGATORS**: Dr. Seif Shekalaghe, Dr. Frank Mosha: KCMC, PO Box 3010, Moshi, Tanzania; Dr. Chris Drakeley, JMP, PO Box 2228, Moshi, Tanzania; Prof Dr. Robert Sauerwein, Teun Bousema (UMCN, Medical Microbiology 268, 6500 HB Nijmegen).

**PARTICIPATION INFORMATION**: We would like your child to participate in a medical research study. It is very important that you understand the following general principles, which apply to all participants in our studies

1. Participation is entirely voluntary.
2. Persons may withdraw from participation in this study or any part of the study at any time. Refusal to participate will not affect your treatment.
3. After you read the explanation, please feel free to ask any question that will allow you to understand clearly the nature of the study.

**INTRODUCTION**:

Malaria is caused by parasites that are transmitted by mosquito bites and can be cured by early treatment with medicine. Sometimes certain parasites remain in your blood after taking the medicine. These parasites cannot make you ill but when a mosquito bites you, it can still pass the disease on to other people who will then also get malaria. We want to study the effect of several medicine on the transmission of malaria, so that we can select the best medicine to slow down the spread of malaria.

**PROCEDURES TO BE FOLLOWED**:

We will take a medical history, examine your child and then obtain a small amount of blood by pricking his/her finger. Your child will receive treatment for malaria by a qualified doctor and we will provide paracetamol to reduce the fever.

Your child will be assigned to one of 2 groups to receive malaria treatment. All these drugs are already used for malaria treatment and are safe to be used.

1. A combination of the usual drug (sulphadoxine-pyrimethamine) for one day and artesunate for three days
2. A combination of the usual drug (sulphadoxine-pyrimethamine) for one day and artesunate for three days and primaquine for one day.

We will then ask you and your child to return to the clinic for evaluation 1, 2, 7, 14, 28 and 42 days later. A clinician will administer treatment during the clinic visits. On all visits we will ask you questions on the child's health, take his/her temperature and examine his/her blood for presence of malaria parasites (blood will be obtained by pricking his/her finger). If on any of these visits the clinician thinks that your child requires additional health care to cure malaria, the clinician will provide this malaria treatment. You will not be charged for this malaria treatment.

If you accept that we enrol your child to the study, you can bring him/her to our clinic here at the hospital for free malaria treatment during the time you participate in the study.

**RISKS AND DISCOMFORTS:** The risks to your child from participating in this study are negligible. There is possibility of mild discomfort and bruising at the site from where blood is obtained. Your child will be carefully monitored for any unforseen side effects.

**DURATION OF VOLUNTEER'S PARTICIPATION:** 42 days

**BENEFITS:** Participants will receive free malaria treatment during participation in the study.

**NUMBER OF VOLUNTEERS IN THE STUDY:** 100 during 2 months.

**ASSURANCE OF CONFIDENTIALITY OF VOLUNTEER'S IDENTITY:** Records relating to your child's participation in the research will remain confidential. The name of your child will not be used in any report resulting from this study. You will receive a copy of this consent form.

**CIRCUMSTANCES UNDER WHICH YOUR CHILD'S PARTICIPATION MAY BE TERMINATED WITHOUT YOUR CONSENT:**

1. Health conditions under which participation would be dangerous.
2. Other conditions which might occur that would make continued participation detrimental to his/her own health.

**MEDICAL CARE FOR INJURY OR ILLNESS:** Your child will be entitled to free malaria treatment during your participation in this research project.

**FOR INFORMATION OR ANSWERS TO QUESTIONS CONCERNING YOUR RIGHTS AS A RESEARCH SUBJECT YOU MAY CONTACT:** Dr. Frank Mosha, Dr. Seif Shekalaghe, KCMC, PO Box 3010 Moshi, Tanzania. Mobile phone: 0745470472

**IF THERE IS ANY PORTION OF THIS CONSENT EXPLANATION SHEET THAT YOU DO NOT UNDERSTAND, ASK THE INVESTIGATOR BEFORE SIGNING.**

I acknowledge receipt of this Informed Consent Explanation.

# *Child's name ______________________________________________________*

Parent's/guardian signature:____________________ Date_______________

# *Parent's/guardian name:____________________________________________*

Witness's Signature: ____________________________ Date: ______________

**INFORMED CONSENT AGREEMENT**

# Study Title: The gametocytocidal activity of sulphadoxine-pyrimethamine *plus* artesunate followed by a single dose of primaquine.

I, ____________________________________________(PARENT/GUARDIAN'S NAME), having full capacity to consent for my child ____________________________________ (CHILD'S NAME), do hereby consent to his/her participation in the research study entitled “The gametocytocidal activity of sulphadoxine-pyrimethamine *plus* artesunate followed by a single dose of primaquine.”, under the principal investigator Dr. Seif Shekalaghe. The implications of my voluntary participation, the nature, duration and purpose; methods and means by which it is to be conducted; and the inconveniences and hazards which may reasonably be expected have been explained to me by _________________________, and are set forth in the Informed Consent Explanation, which I have signed. I have been given an opportunity to ask questions concerning this investigational study, and any such questions have been answered to my full and complete satisfaction. If there are any further questions that may arise, I may contact Dr. Seif Shekalaghe (Kilimanjaro Christian Medical Centre, KCMC) at 0745470472. I understand that I may at any time during the course of this study revoke my consent and withdraw my child from the study without prejudice; however, I may be requested to have my child undergo further examinations if, in the opinion of the physician, such examinations are necessary for his/her well being.

# *Child's name ______________________________________________________*

Parent's/guardian's signature:_____________________ Date_______________

# *Parent's/guardian's Printed Name:____________________________________*

Witness's Signature: ____________________________ Date: ______________

Witness's Printed Name:_____________________________________________

**MAELEZO YA MAKUBALIANO (MAARIFIANO)**

(Yasomwe na Mshiriki ajibu kwa lugha anayoielewa vyiema).

**KICHWA:** **The gametocytocidal activity of sulphadoxine-pyrimethamine *plus* artesunate followed by a single dose of primaquine.**

**VYIO**: Kilimanjaro Christian Medical Centre (KCMC), Moshi, Tanzania; Joint Malaria Programme (JMP), Moshi, Tanzania; University Medical Centre Nijmegen (UMCN).

**WACHUNGUZI WAKUU**: Dr. Seif Shekalaghe, Dr. Frank Mosha: KCMC, PO Box 3010, Moshi, Tanzania; Dr. Chris Drakeley, JMP, PO Box 2228, Moshi, Tanzania; Prof Dr. Robert Sauerwein, Teun Bousema (UMCN, Medical Microbiology 268, 6500 HB Nijmegen).

**UJUMBE KWA WASHIRIKI**: Tungependa mwanao auzike kwenye masomo ya uchunguzi wa madawa. Kuna manufaa yako kuelewa maadili na mahitayi ya kawaida kwa kila mshiriki.

1. Kushiriki ni kujitolea kwa ihali
2. Unaweza kujiondoa kwenye hii shughuli au sehemu yake. Kujiondoa hakutayadhulu matibabu yako.
3. Baada ya kusoma haya Maelezo, una uhuru wa kuuliza swali lolote ili uwe mwenye keulewa kikamilifu.

**UTANGULIZI:**

Ugonjwa wa malaria husababishwa na chembe chembe zinazoenezwa na umbu anapokuuma, na hutibiwa na dawa, Kufikia sasa dawa nyingi zimekosa kumaliza hizi chembe chembe. Hizi chembe chembe hazina uwezo wakukufanya uwe mgonjwa lakini Umbu anapokuuma ana uwezo wa kumwambukiza mtu mwingine malaria anapomuuma. Tunahitaji kuchungua madawa tofauti kwa uambukizaji wa malaria ili tuweze kupata yenye kufaa zaidi kwa kuzuia kuenezwa kwa ugonjwa na zaidi kutoweza kufanya matibabu, kwa upande wenu.

**MAELEZO YA MAAGIZO**

Tutahitaji historia ya mwanao, tumkague na kuchukua tone la damu kwenye kidole chake. Mwanao atapokea matibabu ya malaria kutoka kwa daktari aliye fuzu na kisha atapewa dawa ya paracetamol kuzuia maumivu ya choto. Mwanao atakua kwenye kundi moja kati ya makundi mbili ya kupokea matibabu ya malaria. Dawa hizi zote tayari ni zile zinazo tumika kwa kutibu malaria na zimekubalika.

1. Mchanganyiko wa dawa ya kawaida (Sulphadoxine-pyrimethamine) kwa siku moja na artesunate kwa siku tatu.
2. Mchanganyiko wa dawa ya kawaida (Sulphadoxine-pyrimethamine) kwa siku moja na artesunate kwa siku tatu na primaquine kwa siku moja.

Kisha tutakuomba umrudishe mwanao kwa hospitali kwa udadizi kwa siku ya 1, 2, 7, 14, 28 na ya 42. Muuguzi atampa dawa vipindi vyote mtakapokuja hospitalini. Kwa huu muda wote tutajulie mwanao hali yake kiafya, kwa kupima choto mwilini na chembe za malaria kwenya damu (damu itatolewa tone kwenye kidole). Ikiwa muuguzi ataonelea mwanao anahitaji dawa zaidi baada ya kumkagua ili kuimaliza malaria itambidi kufanya hivyo. Hutahitajika kugaramia haya matibabu ya malaria.

Ukikubali kumwandikisha mwanao kwenye huu uchunguzi, utamleta kwa hospitali yetu kwa matibabu ya bure ya malaria wakati huu wa masomo.

**HATARI NA MAUMIFU:** Hakutakuwa na hatari yoyote mwanao atakapokuwa anahusika kwenye huu uchunguzi. Kunauwezekano wa uchungu kidogo kwenye sehemu damu itatolewa.

**MUDA WAKUSHIRIKI:** Siku arobaini na mbili.

**FAIDA:** washiriki watapata matibabu ya malaria ya bure wakati wa huu uchunguzi.

**KIWANGO CHA WASHIRIKI:** watu 100 (mia moja) kati ya miaka (2-3) .

**AHADI YA KUWEKA SIRI KWA MAJINA YA MASHIRIKI:** Nakala za kuonyesha kushiriki kwa mwanao kwenye uchunguzi zitahifadhiwa kwa ujasili. Jina la mwanao halitatumika kwenye ujumbe wowote unaohusu uchunguzi huu. Utapokea paja la karatasi hii ya makubaliano.

**SEHEMU MWANAO ANAWEZA KUONDOLEWA KWA HUU UCHUNGUZI BILA WEWE KUARIFIWA:**

1. Ikiwa afya yake itakuwa kwenye hatari akishirikishwa.
2. Matokeo mengine ya kiafya ambayo yatasababisha matatizo kwa afya yake ikiwa ataendelea kushiriki

**HUDUMA ZA TIBA PANAPO NA AJALI AU UGONJWA:** Mwanao atapokea matibabu ya bure ya malaria kipindi atakuwa anashiriki.

**UJUMBE AU MAJIBU YA MASWALI JUU YA HAKI ZAKO KAMA MSHIRIKI KWENYE HUU UCHUNGUZI UTAYAPATA KWA:** Dr. Frank Mosha, Dr. Seif Shekalaghe, KCMC, PO Box 3010 Moshi, Tanzania. Mobile phone: 0745470472

IKIWA KUNA SEHEMU YA HII KARATASI YA MAAFIKIANO AMBAYO HUELEWI, ULIZA MCHUNGUZI KABLA YA KUWEKA KIDOLE.

Ninakubali kupokea maelezo ya makubaliano.

# *Jina la mtoto: ________________________________________________________________*

Sahihi ya mzazi/mlezi:__________________ Tarehe______________________

# *Jina la mzazi/mlezi (Japisha):_________________________________________*

Sahihi ya mshahidi ____________________ Tarehe: ______________________

**MAELEZO YA MAKUBALIANO (MAAFIKIANO)**

**KICHWA:** **The gametocytocidal activity of sulphadoxine-pyrimethamine *plus* artesunate followed by a single dose of primaquine.**

Mimi, ________________________________________(JINA LA MZAZI/MLEZI), nina uwezo wa Maafikiano kwa mtoto wangu ____________________________ (JINA LA MTOTO), nakubali ausike kwenye uchunguzi wa ‘The gametocytocidal activity of sulphadoxine-pyrimethamine *plus* artesunate followed by a single dose of primaquine’. Jina la mkaguzi (mchunguzi) mkuu Dr. Seif Shekalaghe. Matokeo ya kujitolea kwangu kushiriki, na aina, muda na sababu ; na namna ya kufanywa na mikozi na tahadhari zenye sinaweza kutarajiwa nimeelezewa na _________________________, na zimearifiwa kwenye karatasi ya maafikiano ambayo tayari nimeweka sahihi yangu. Nimepewa nafasi ya kuuliza maswali kuhusu huu uchunguzi, na maswali hayo yote nimeelezewa na kuridhika. Kama kutatokea maswali mengine ninaweza kumuuliza Dr. Seif Shekalaghe (Kilimajaro Christian Medical Centre, KCMC) kwa 0745470472 Ninaelewa kwamba wakati wowote wa huu uchunguzi ninaweza kujiondoa pamoja na mtoto wangu bila kasheshe yeyote; Si hoja, ninaweza kuombwa mwanangu afanyiwe uchunguzi zaidi kama,, kulingana na Muuguzi, uchunguzi huo unahitajika kwa manufaa ya mwanangu.

# *Jina la mtoto _____________________________________________________*

Sahihi ya mzazi/mlezi:_____________________________Tarehe:___________________ Jina la mzazi/mlezi: ________________________________________________

# *Sahihi ya mshahidi: ________________________________________________*

Jina la mshahidi:___________________________________________________

CASE RECORD FORM

**IDNR**: ___ ___ ___

Patient identification

NAME:

NAME FATHER:

NAME MOTHER:

ADDRESS:

IDNR:

Recorded at health clinic ______________ at ___ __ / __ __ / __ __ (dd/mm/yy)

| *1. ADMISSION: does the patient meet entry criteria? Answer questions below* | | | |
| --- | --- | --- | --- |
| **Inclusion (Y/N)** | | *Exclusion (Y/N)* | |
| age 6 months and above |  | danger signs or severe malaria |  |
| Mono-infection with *P. falciparum*, density 500-100.000 P/μl/ malariae mixed infection |  | Haemoglobin <8g/dl |  |
| Temperature >37.5˚C and <39.5˚C, or a history of fever in the previous 24 hours |  | presence of diseases other than malaria causing febrile conditions /severe malnutrition |  |
| Resident of the research area (Able to complete follow-up)? |  | Reported history of adverse reactions to study drug |  |
| Understanding of the procedures of the study and willing to participate. Consent from patient or guardian/parent |  | Reported use of SP in the previous two weeks |  |
|  | **(Y/N)** |  | (Y/N) |
| **All inclusion criteria answered YES?** |  | All exclusion criteria answered NO? |  |
| **INCLUDED?** |  | CONSENT FORM ATTACHED? |  |
| If the patient is not included in the study, refer him/her to the clinician for further examination and treatment | | | |

| **2. REGISTRATION: If all inclusion criteria are answered YES & all exclusion criteria NO, proceed** |
| --- |
| *Assign the patient a study number and fill out the patient identification data above**Fill out demographic information below* |

| **3. DEMOGRAPHY** | | | |
| --- | --- | --- | --- |
| **Gender (M/F)** |  | **Age in months** |  |
| **Weight (kg)** | ─ ─ , ─ | **Date of Birth (dd/mm/yy)** | ─ ─ / ─ ─ / ─ ─ |

| **4. TREATMENT ALLOCATION (tick one) :** | |
| --- | --- |
| Sulphadoxine-Pyrimethamine + Artesunate  Sulphadoxine-Pyrimethamine + Artesunate + Primaquine |  |
|  |

| **Principal Investigator’s name** | **Signature** | **Date** |
| --- | --- | --- |
|  |  |  |

| **5. TREATMENT** | | | | | | |
| --- | --- | --- | --- | --- | --- | --- |
|  | **Scheduled treatment** | | | | | |
| ***Day of study*** | ***D0*** | ***D1 (24hr)*** | | | ***D2 (48hr)*** | |
| Date (dd/mm/yy) | _ _ / _ _ / _ _ | _ _/_ _/_ _ | | | _ _/_ _/_ _ | |
| Study treatment administered? (Y/N) |  |  | | |  | |
| 24hr clock time treatment given [hh : mm] | _ _ : _ _ | _ _ : _ _ | | | _ _ : _ _ | |
| *If treatment not given, specify reason* |  |  | | |  | |
| *If study treatment permanently withdrawn, specify indication for early termination (tick one)* | | | | | | |
| Parasitaemia increased > 25% from baseline and no clinical improvement at 48h | | | | | |  |
| Any sign of severe malaria, as per WHO definition, or a requirement for parenteral treatment | | | | | |  |
| Any severe adverse event requiring treatment withdrawal | | | | | |  |
| Patient’s or parent/guardian’s decision | | | | | |  |
| Rescue treatment administered? (if Yes, provide details below) | | | | | |  |
| **6. Other therapies (other than paracetamol, cough syrup and vitamins)** | | | | | | |
| Generic name of medication | **Dose** | | Start date | End date | | |
|  |  | |  |  | | |
|  |  | |  |  | | |

| 7. ASSESSMENTS | Treatment days | | | | Follow-up | | | | | | | | | |
| --- | --- | --- | --- | --- | --- | --- | --- | --- | --- | --- | --- | --- | --- | --- |
| Date(dd/mm/yy) | _ _/_ _/_ _ | _ _/_ _/_ _ | | _ _/_ _/_ _ | _ _/_ _/_ _ | | | _ _/_ _/_ _ | | _ _/_ _/_ _ | _ _/_ _/_ _ | | _ _/_ _/_ _ | |
| Day of study | **D0** | **D1** | | **D2** | **D3** | | | **D7** | | **D14** | **D28** | | **D42** | |
| Was the child seen (Y/N) |  |  | |  |  | | |  | |  |  | |  | |
| **Samples collected** | **Label all samples with IDNR and day of follow-up** | | | | | | | | | | | | | |
| Haemoglobin (Y/N) |  |  |  | |  | |  | |  | | |  | |  |
| Thick/thin smear (Y/N) |  |  |  | |  | |  | |  | | |  | |  |
| NASBA sample (Y/N) |  |  |  | |  | |  | |  | | |  | |  |
| Filter paper (Y/N) |  |  |  | |  | |  | |  | | |  | |  |
| *Body Temperature* | _ _ , _ºC | _ _ , _ºC | _ _ , _ºC | | _ _ , _ºC | | _ _ , _ºC | | _ _ , _ºC | | | _ _ , _ºC | | _ _ , _ºC |
| *Parasitology* | **P. falciparum only, mixed infections will be excluded** | | | | | | | | | | | | | |
| Asexual / 200WBCs |  |  |  | |  | |  | |  | | |  | |  |
| *Gametocytes / 500 WBCs* |  |  |  | |  | |  | |  | | |  | |  |
| *Signs & Symptoms* | **If the child is clinically ill, refer to clinician for examination** | | | | | | | | | | | | | |
| Day of study | **D0** | **D1** | **D2** | | | **D3** | **D7** | | **D14** | | | **D28** | | **D42** |
| Headache (Y/N) |  |  |  | | |  |  | |  | | |  | |  |
| Diarrhoea (Y/N) |  |  |  | | |  |  | |  | | |  | |  |
| Abdominal pain (Y/N) |  |  |  | | |  |  | |  | | |  | |  |
| Weakness (Y/N) |  |  |  | | |  |  | |  | | |  | |  |
| **Other, specify and mention the day** |  | | | | | | | | | | | | | |

| **8. TREATMENT OUTCOME (circle)** | **ACR** | **ETF** | **LTF** | **Excluded** | **Lost to follow-up** |
| --- | --- | --- | --- | --- | --- |
